# Accumulation of invariant NKT cells with increased IFN-γ production in persistent high-risk HPV-infected high-grade cervical intraepithelial neoplasia

**DOI:** 10.1186/s13000-015-0254-8

**Published:** 2015-04-02

**Authors:** Ting Hu, Pei Yang, Hongmei Zhu, Xinlian Chen, Xiaoyan Xie, Mei Yang, Shanling Liu, He Wang

**Affiliations:** Department of Obstetrics and Gynecology, West China Second University Hospital, Sichuan University, No. 20, Section 3, Renming Nan Road, Chengdu, 610041 China; Laboratory of Cell and Gene Therapy, West China Institute of Women and Children’s Health, West China Second University Hospital, Sichuan University, No. 20, Section 3, Renming Nan Road, Chengdu, 610041 China; Laboratory of Genetics, West China Institute of Women and Children’s Health, West China Second University Hospital, Sichuan University, No. 20, Section 3, Renming Nan Road, Chengdu, 610041 China; Key Laboratory of Obstetric & Gynecologic and Pediatric Diseases and Birth Defects of Ministry of Education, No. 20, Section 3, Renming Nan Road, Chengdu, 610041 China

**Keywords:** Human papillomavirus, Cervical intraepithelial neoplasia, Invariant natural killer cells, Gamma interferon, CD3+ T cells

## Abstract

**Background:**

Persistent high-risk human papillomavirus (HR-HPV) infection has been implicated in the development of high-grade cervical intraepithelial neoplasia (CIN) and cervical cancer. Invariant natural killer T (iNKT) cells produce large amounts of cytokines to regulate immune responses. However, the role of iNKT cells in human persistent HPV-infected cervical tissues is unknown.

**Methods:**

In our study, 201 patients with diagnoses ranging from normal ectocervical tissue to CINIII from June 2010 to May 2012 were enrolled. HPV DNA and HPV types were detected using the hybrid capture-2 HPV DNA test. Flow cytometry was used to investigate iNKT and CD3+ T cell infiltration into cervical tissues. Real-time quantitative reverse transcription-polymerase chain reaction was used to study IFN-γ expression and immunohistochemistry was used to determine CD3+ T cell distribution.

**Results:**

A significant increase in iNKT cells was observed in HPV-positive cervical tissues (*p* < 0.05), especially in CINII-III (*p* < 0.01). IFN-γ expression was also increased in HPV-positive cervical tissues (*p* < 0.05). CD3+ T cells were detected among both epithelium and stromal layers in cervical tissues, and the percentage of CD3+ T cells in HPV-positive cervical tissues was similar to that in HPV-negative cervical tissues (*p* > 0.05).

**Conclusions:**

The iNKT cell aggregation in cervical tissues during the progression from HPV infection to CIN indicates that iNKT cells might play an important role in suppressing immunity. IFN-γ expression could also be related to the HPV infection status. Preventing the accumulation or functioning of iNKT cells in cervical tissues may be a viable method to prevent the development of CIN.

**Virtual slides:**

The virtual slides for this article can be found here: http://www.diagnosticpathology.diagnomx.eu/vs/2521874671514142

## Background

Cervical cancer is the third most common cancer worldwide in females, with approximately 529,800 new cases each year, nearly 85% of which occur in developing countries [1]. Infection with human papillomavirus (HPV) is a major cause of cervical cancer. After infection with HPV, precursor lesions precede the development of cervical cancer; these lesions are referred to as cervical intraepithelial neoplasia (CIN) grades I, II and III and are characterized by a series of histological abnormalities [2]. Low-grade lesions (CINI) usually clear spontaneously. However, high-grade CIN (CINII/III) lesions often persist and may progress to carcinoma [3]. Persistent infection with high-risk (HR) HPV has been implicated in the development of CIN II/III and invasive cervical cancer [4,5]. Infection also triggers a series of immune responses during lesion progression.

Invariant natural killer T (iNKT) cells are a sub-population of T cells characterized by the expression of a restricted αβ T-cell receptor (TCR) repertoire: Vα24-JαQ paired with Vβ11 in humans. iNKT cells also co-express natural killer (NK) cell receptors such as NK1.1 [6]. Distinct from conventional T cells, iNKT cells specifically recognize a glycolipid antigen α-galactosylceramide (αGalCer) presented by the non-classical class I-like MHC molecule CD1d. CD1d-recruited iNKT cells rapidly produce large amounts of Th1 and Th2 cytokines, including gamma interferon (IFN-γ), interleukin-4 (IL-4), IL-10 and IL-13, which protect against viral infections and cancers [7,8]. iNKT cells also suppress self-reactive immune responses, preventing autoimmunity [9,10] and contributing to transplant tolerance induction [11,12].

Several recent studies have demonstrated the pivotal anti-tumor effects of iNKT cells and have reported reduced numbers of iNKT cells in patients with malignant diseases, including colon cancer, head and neck cancer, breast cancer, renal cell cancer and melanoma [13]. In a mouse model [14], iNKT cells were attracted to a grafted HPV16-E7-expressing epithelial hyperplasia instead of HPV-negative tissues and expressed IFN-γ. iNKT cells, the major source of IFN-γ, had the paradoxical capacity to locally suppress immune effector mechanisms after specifically being attracted to persistent HR-HPV-infected lesions.

Thus far, nothing is known about the specific role of iNKT cells in regulating local cervical immunity in humans. In this study, we asked whether iNKT cells play a role in persistent human HPV-infected cervical tissues. We demonstrated that in the progression from HPV infection to CIN, iNKT cell accumulation might play an important role in suppressing immunity. Further, IFN-γ expression could also be related to HPV infection status. Preventing the accumulation or functioning of iNKT cells in cervical tissues may be a viable way to prevent the development of CIN.

## Methods

### Patients

All clinical specimens were obtained from the outpatient department of gynecology in West China Second University Hospital, Sichuan University. Written informed consent was obtained from all patients. Patients with abnormal Pap tests who needed to be diagnosed by biopsy using a colposcope were eligible for this study. All patients were older than 25 years old; patients included in this study had not received any prior cervical treatment within 12 months from study enrollment and did not suffer from other infections (such as herpes simplex virus [HSV] or chlamydia); malignancies and gravidas were excluded. This study was approved by the Medical Ethics Committee of West China Second University Hospital, Sichuan University (approval number 2009-044).

### Detection of HPV DNA in cervical specimens

HPV DNA specimens were collected with cervical brushes (Digene, Gaithersburg, Maryland, USA) prior to the application of acetic acid and iodine using a colposcope. HPV DNA was analyzed using the Digene Hybrid Capture® 2 High-Risk HPV DNA Test kit (Qiagen, Gaithersburg, Maryland, USA) according to the manufacturer’s instructions. Thirteen HR-HPV types were detected, including types 16, 18, 31, 33, 35, 39, 45, 51, 52, 56, 58, 59 and 68.

Five hundred microliters of denaturation reagent was added to the specimen transport medium, and the sample was placed in a 65°C water bath for 45 minutes. Then, 75 μl of the mixture and 25 μl of the high-risk HPV probe were transferred to a hybridization microplate, vortexed for 3 minutes, and incubated in a 65°C water bath for 30 minutes. The entire contents were pipetted into a capture microwell and vortexed for 60 minutes at room temperature (RT). After discarding the liquid from the wells, 75 μl of detection reagent 1 was added to the wells for 30 minutes at RT and discarded again. After washing the capture wells with wash buffer 6 times, 75 μl of detection reagent 2 was added to the wells for 15 minutes in the dark. The negative high-risk calibrator and the quality control reagent were tested simultaneously. Finally, the results were analyzed using the Digene assay analysis software DML 2000. Signal strengths with relative light units (RLU)/cutoff (CO) ratios ≥1.0 were considered positive.

### Biopsy samples

The biopsy samples were obtained under a colposcope according to a standard protocol that included conventional visual assessment, the application of 5% acetic acid and iodine, the recognition of the transformation zone and the identification of the abnormal area. In each patient, one biopsy was taken from a suspected CIN lesion of the cervix, and another biopsy was taken from a normal area. We harvested the suspected CIN lesion from the most typical and severe parts of the lesions under a colposcope to avoid the possibility of different grades presented in the same specimen to the greatest extent. Each biopsy sample was divided into 2 parts for histopathological diagnosis in the pathology department and for laboratory research. The histopathological diagnosis was reviewed by two senior experts from pathology department in our hospital. The biopsy samples for flow cytometry were transported in media at 4°C and analyzed within two hours. The biopsy samples for real-time quantitative reverse transcription-polymerase chain reaction (RT-PCR) were immediately frozen in liquid nitrogen. The biopsy samples for immunohistochemistry (IHC) were immersed in 10% formalin for 24 hours and embedded in paraffin.

### Regents and flow cytometry

Anti-human monoclonal antibodies (Ab) to CD3 (Clone UCHT1), TCR Vα24 (Clone Cl5), TCR Vβ11 (Clone C21), IgG1 (Clone 679.1Mc7) and IgG2a (Clone 7 T4-1 F5) were purchased from Beckman Coulter (Fullerton, California, USA). The FcR Blocking Reagent was purchased from Miltenyi Biotec (Friedrich-Ebert-Straße, Bergisch Gladbach, Germany). The Live/Dead Near-IR Fixable Dead Cell staining kit was purchased from Invitrogen (Eugene, Oregon, USA).

The cervical tissue was cut into the smallest pieces possible in ice-cold media at 4°C. The tissue suspension was transferred to a 70-μm cell strainer. After centrifuging the cell suspensions at 30 g for 3 minutes to remove cell aggregates and debris, the supernatant was then centrifuged at 400 g for 5 minutes at 4°C. The cervical tissue cells were used for flow cytometry by discarding the supernatant. The resuspended cells were divided into 2 tubes with an optimal volume of 100 μl for 510^5^ - 110^6^ cells and were stained with anti-CD3, anti-TCR Vα24 and anti-TCR Vβ11 antibodies or anti-CD3, IgG1 and IgG2a as isotype controls for 30 minutes at 4°C in the dark. Prior to the antibody staining of cells, FcR block and viability stains were added to each tube for 30 minutes on ice in the dark. Negative controls and single-color compensation controls were prepared simultaneously. Stained cells were analyzed on a FACSCalibur flow cytometer (BD Biosciences; San Jose, California, USA).

### Real-time quantitative RT-PCR

The cervical tissue was ground as finely as possible in liquid nitrogen using a pestle. Total RNA was extracted using TRIzol reagent according to the manufacturer’s instructions (Invitrogen). To avoid contamination with genomic DNA, cDNA was synthesized from total RNA using the PrimeScript® RT reagent Kit with gDNA Eraser (TaKaRa Biotechnology, Dalian, China) and was stored at -80°C.

Real-time quantitative PCR reactions were performed in a total volume of 50 μl in the presence of 5 μl of cDNA, 300 nM of forward and reverse PCR primers and 25 μl of iQ SYBR Green Supermix (BIO-RAD). Each cDNA sample was analyzed in duplicate using an ABI Prism 7300 Sequence Detector (Applied Biosystems) under the following conditions: an initial step of 3 minutes at 95°C; 40 cycles of 15 seconds at 95°C, 15 seconds at 55°C and 30 seconds at 60°C; and a final melting-curve step of 15 seconds at 95°C, 30 seconds at 60°C and 15 seconds at 95°C to prevent nonspecific fluorescence derived from unintentional products such as primer-dimers. SYBR Green real-time PCR primers for IFN-γ, CD3 and glyceraldehyde-3-phosphate dehydrogenase (GAPDH) were as follows: IFN-γ [15], forward 5′-GAA TTG GAA AGA GGA GAG TGA CAG A-3′, reverse 5′-GTC TCC ACA CTC TTT TGG ATG CT-3′; CD3 [16], forward 5′-TCG CCA GTC GAG AGC TTC A-3′, reverse 5′-GGG CTG GTA GAG CTG GTC ATT-3′; and GAPDH [17], forward 5′-CCA CCC ATG GCA AAT TCC-3′, reverse 5′-GAT GGG ATT TCC ATT GAT GAC A-3′. After the reactions, standard curves and cycle threshold (Ct) values were determined. All Ct values were normalized to the housekeeping gene GAPDH to adjust for unequal amounts of RNA.

### Immunohistochemistry

The formalin-fixed, paraffin-embedded cervical tissue blocks were sectioned at 4-μm thickness and mounted onto SuperFrost/Plus glass slides. The slides were deparaffinized and rehydrated and then incubated in a 3% H_2_O_2_ solution in methanol at RT for 10 minutes to block endogenous peroxidase activity. The sections were incubated with 10 mM citrate buffer (pH 6.0) at 95°C for 10 minutes to unmask the antigen epitopes and with normal goat serum (NGS) in phosphate-buffered saline (PBS) at RT for 1 hour to prevent non-specific antibody binding. The primary antibody, mouse monoclonal anti-human CD3 (Clone PS1, ZSGB-BIO), was applied at a 1:50 dilution to sections and incubated in a humidified chamber at 4°C overnight. For primary antibody detection, specific goat polyclonal antibodies were used at a dilution of 1:200 for 30 minutes at RT. After washing in PBS, streptavidin-horseradish peroxidase (Sav-HRP) conjugates were applied to the sections in the dark at RT for 30 minutes, followed by a diaminobenzidine (DAB) substrate solution to reveal the color of the antibody staining. For negative controls, sections were stained with PBS instead of the primary Ab. Normal human normal lymph nodes were used as a positive control. All slides were counterstained with hematoxylin. Positive cell numbers were analyzed by microscopic counting (400×) from 10 randomly selected areas.

### Statistical analysis

The statistical analysis was performed using the SPSS software, version 13.0. Results groups are shown as the means ± standard deviations (SDs). Comparisons between groups were performed using two-tailed Student’s *t* tests and analysis of variance with Levene’s test. If the distribution was extremely skewed, a nonparametric two-tailed Mann-Whitney U test was used. Comparisons among multiple groups were performed with one-way analysis of variance (ANOVA) followed by Fisher’s protected least significant difference (PLSD) post hoc test. When the *P* value was <0.05, differences were considered significant.

## Results

### Patient characteristics

From June 2010 to May 2012, a total of 201 patients were enrolled in the study. According to the pathological examinations, the biopsies of all of the cervical tissues were diagnosed as normal ectocervical tissue (NCT), chronic cervicitis, CINI, CINII or CINIII. In the study, 134 patients were classified as HPV-positive (66.7%) by HC-2, 67 of whom were diagnosed with high-grade CIN by a pathologist. In the HPV-negative group, none of the subjects were diagnosed with high-grade CIN. The patient characteristics are summarized in Table 1. There were 72 patients included in the flow cytometry test, 62 patients in the RT-PCR test and 67 patients in the IHC test. The patient classification for each test is presented in Table 2.

**Table 1:** **Patient characteristics**

	**HPV-positive group**	**HPV-negative group**
Total number entered	134	67
Median age (range)	38.2 ± 7.9	36.9 ± 8.7
Pathological results, (NCT/cervicitis/CINI/II/III)	35/21/20/25/33	27/21/19/0/0

**Table 2:** **Patient classification**

	**HPV-positive group**			**HPV-negative group**		
	**FCM**	**RT-PCR**	**IHC**	**FCM**	**RT-PCR**	**IHC**
NCT	13	12	10	9	8	10
cervicitis	7	8	6	7	7	7
CIN I	6	7	7	8	5	6
CIN II	8	9	8	0	0	0
CIN III	14	6	13	0	0	0

### Distribution of CD3+ T cells in cervical tissues

**Figure 1:**
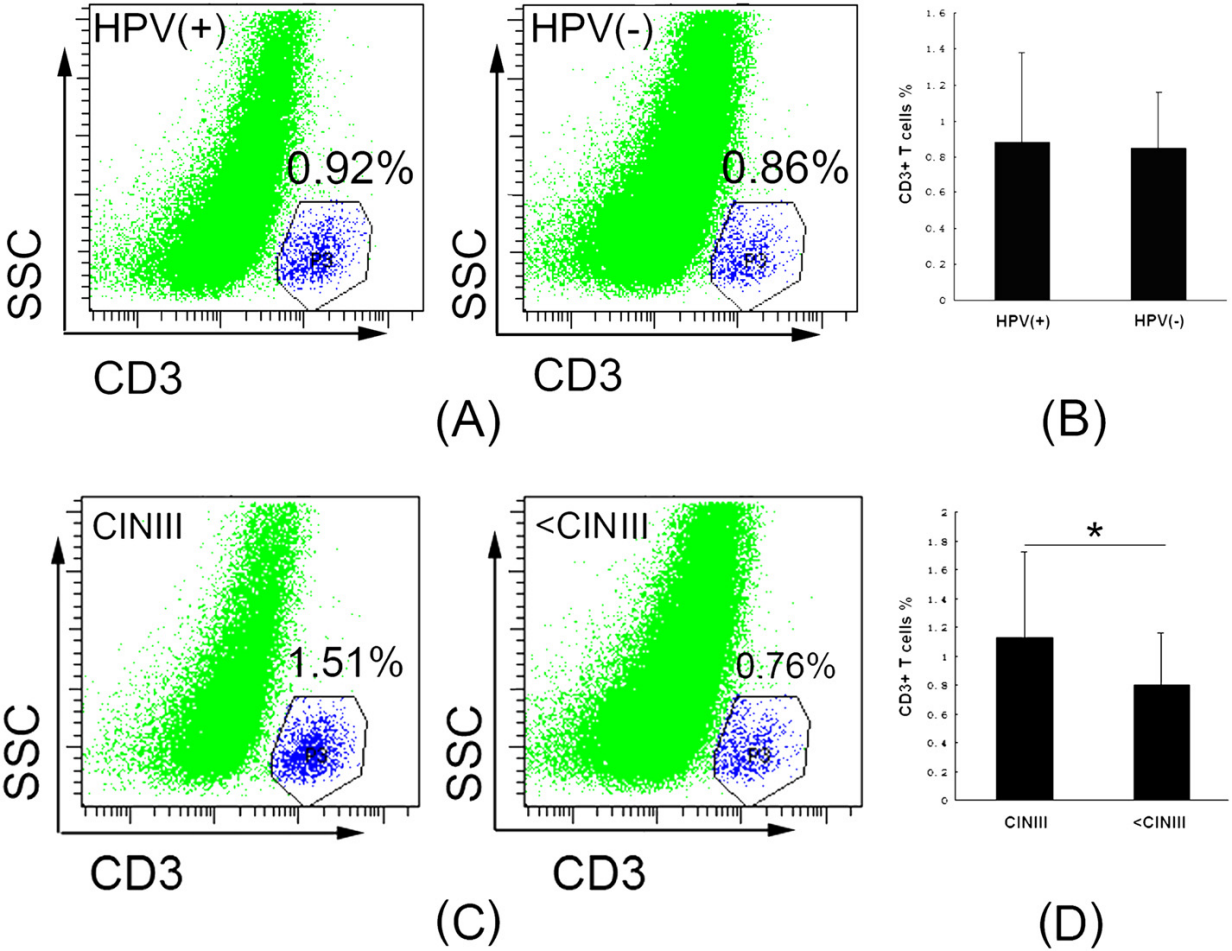
**The percentage of CD3+ T cells in live cells of human cervical tissues in the HPV-positive group is similar to that in the HPV-negative group, but significantly increased in CINIII cervical tissues. A**, Flow cytometry plots of CD3+ T cells in live cells of HPV-positive and HPV-negative groups, as detected by CD3-APC staining; **B**, The bar graph shows CD3+ T cells as percentages of live cells isolated from HPV-positive and HPV-negative cervical tissues (*p* = 0.775). **C**, Flow cytometry plots of CD3+ T cells in live cells of CINIII and all other < CINIII cervical tissues, as detected by CD3-APC staining; **D**, The bar graph shows CD3+ T cells as percentages of live cells isolated from CINIII and all other < CINIII cervical tissues (*p = 0.045).

We quantified the percentages of CD3+ T cells in the live cell gate (viability stain-negative) in cervical tissues by flow cytometry from an HPV-positive group (*n* = 48) and an HPV-negative group (*n* = 24). The proportion of CD3+ T cells in the HPV-positive group was similar to that in the HPV-negative group (mean, 0.8845% *vs*. 0.8483%, *p* = 0.775) (Figure 1A, B). We then compared the CD3+ T cell percentage in CINIII samples (n = 14) with all of the other samples (n = 58) and found that CD3+ T cell numbers were significantly increased in CINIII cervical tissues (mean, 1.1343% *vs*. 0.7943%, *p* = 0.045) (Figure 1C, D), which is similar to a previous study by Carrero *et al.* [18].

**Figure 2:**
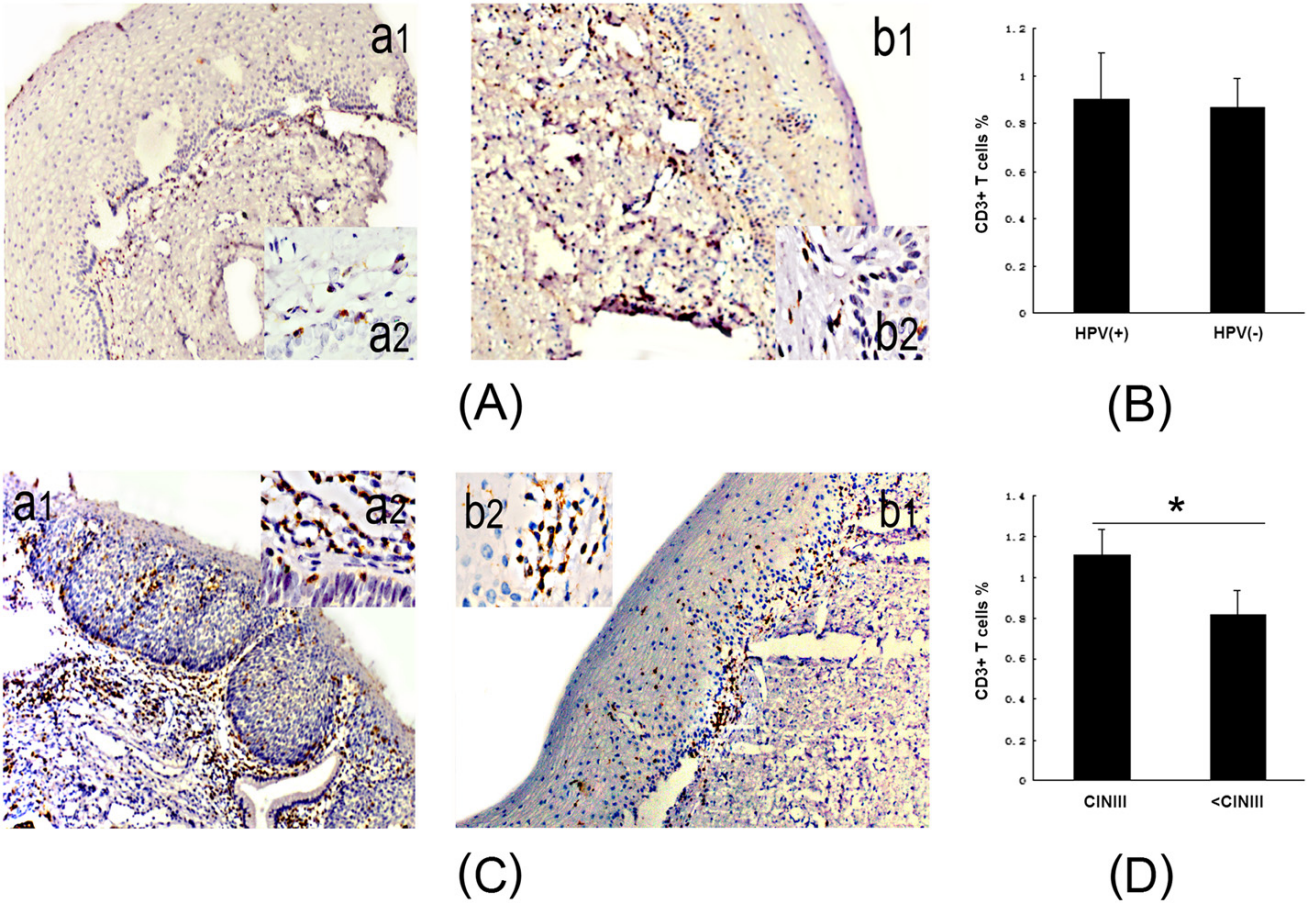
**The distribution of CD3+ T cells in HPV-positive cervical tissues is similar to that in HPV-negative cervical tissues, but significantly increased in CINIII cervical tissues. A**, a1 and a2, IHC of CD3+ T cells in HPV-positive cervical tissues detected by CD3 staining (IHC 10× and 100×); b1 and b2, IHC of CD3+ T cells in HPV-negative cervical tissues detected by CD3 staining (IHC × 10 and × 100). **B**, The bar graph shows CD3+ T cells as percentages of cervical tissues isolated from the HPV-positive and HPV-negative groups (*p* = 0.528). **C**, a1 and a2, IHC of CD3+ T cells in CINIII cervical tissues detected by CD3 staining (IHC 10× and 100×); b1 and b2, IHC of CD3+ T cells in all other < CINIII cervical tissues detected by CD3 staining (IHC 10× and 100×). **D**, The bar graph shows CD3+ T cells as percentages of cervical tissues isolated from CINIII and all other < CINIII cervical tissues (*p = 0.001).

To confirm the distribution of CD3+ T cells in cervical tissues, we immunostained HPV-positive (n = 44) and HPV-negative cervical tissue (n = 23) for CD3. Immunoreactivity with an anti-CD3 Ab was noted in both epithelium and stromal layers from formalin-fixed, paraffin-embedded cervical tissue sections. There were no significant differences in CD3 expression between HPV-positive and HPV-negative tissues (mean, 0.900% *vs*. 0.868%, *p* = 0.528) (Figure 2A, B). Similar to the flow cytometry results, CD3 expression was significantly increased in CINIII samples (n = 13) compared to all of the other samples (n = 54) (mean, 1.108% *vs*. 0.820%, *p* = 0.001) (Figure 2C, D).

### Infiltration of iNKT cells in cervical tissues

**Figure 3:**
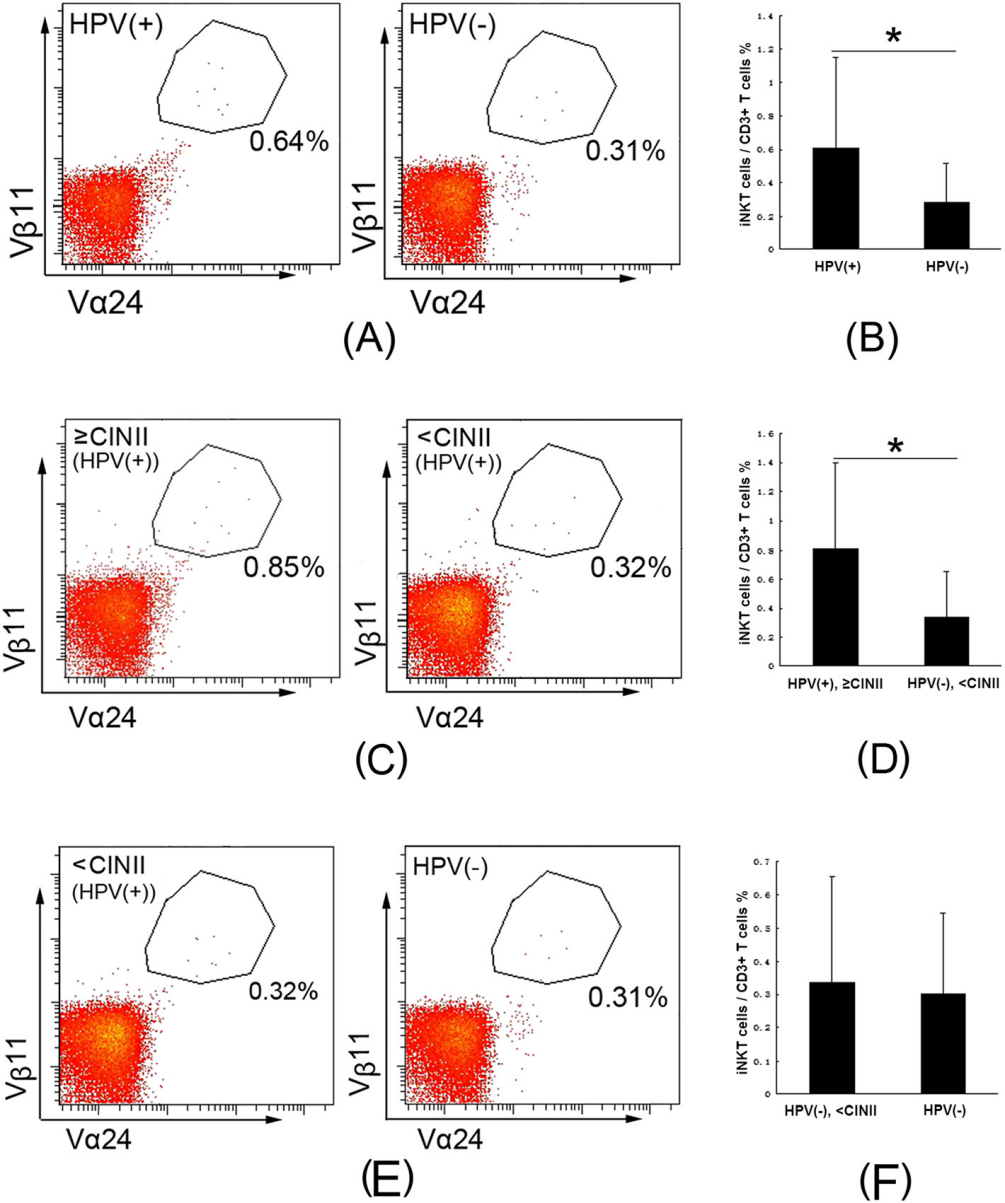
**The proportion of iNKT cells in total CD3+ T cells in HPV-positive cervical tissues is significantly increased, especially increased in ≥ CINII subgroup; and the proportion of iNKT cells to CD3+ T cells in ≥ CINII subgroup is significantly in the HPV-positive group, but in < CINII cervical tissues of the HPV-positive group is similar to that in the HPV-negative group. A**, Flow cytometry plots of iNKT cells in CD3+ T cells in HPV-positive and HPV-negative cervical tissues, as detected by Vα24 and Vβ11 staining; **B**, The bar graph shows iNKT cells as percentages of CD3+ T cells isolated from HPV-positive and HPV-negative cervical tissues (*p = 0.017). **C**, Flow cytometry plots of iNKT cells in CD3+ T cells in the HPV-positive cervical tissues of ≥ CINII and < CINII subgroups, as detected by Vα24 and Vβ11 staining; **D**, The bar graph shows iNKT cells as percentages of CD3+ T cells isolated from HPV-positive cervical tissues of ≥ CINII and < CINII subgroups (*p = 0.001). **E**, Flow cytometry plots of iNKT cells in CD3+ T cells from the ≥ CINII subgroup of HPV-positive cervical tissues and HPV-negative cervical tissues, as detected by Vα24 and Vβ11 staining; **F**, The bar graph shows iNKT cells as percentages of CD3+ T cells isolated from ≥ CINII subgroup of HPV-positive cervical tissues and HPV-negative cervical tissues (*p* = 0.046).

There were no significant differences in CD3+ T cells between the HPV-positive and HPV-negative groups, and iNKT cells are a population of CD3+ T cells. Therefore, to measure the number of iNKT cells in cervical tissues, we used the ratio of Vα24+/Vβ11+ cells to CD3+ T cells as the percentage of iNKT cells. An increased proportion of iNKT cells was observed in the HPV-positive group (n = 48) compared to the HPV-negative group (n = 24) (mean, 0.6062% *vs*. 0.2789%, *p* = 0.017) (Figure 3A, B). Because there is overwhelming evidence that persistent infection with HR-HPV causes high-grade CIN [3,4], we divided the HPV-positive group into 2 groups: a < CINII subgroup, with NCT to low-grade CIN (n = 26), and a ≥ CINII subgroup with high-grade CIN (n = 22). A significantly higher proportion of iNKT cells were detected in the ≥ CINII subgroup compared to the < CINII subgroup (mean, 0.8077% *vs*. 0.3845%, *p* = 0.001) (Figure 3C, D). The proportion of iNKT cells in the < CINII subgroup was similar to that in the HPV-negative group (mean, 0.3845% *vs*. 0.2789%, *p* = 0.466) (Figure 3E, F).

### IFN-γ expression in cervical tissues

**Figure 4:**
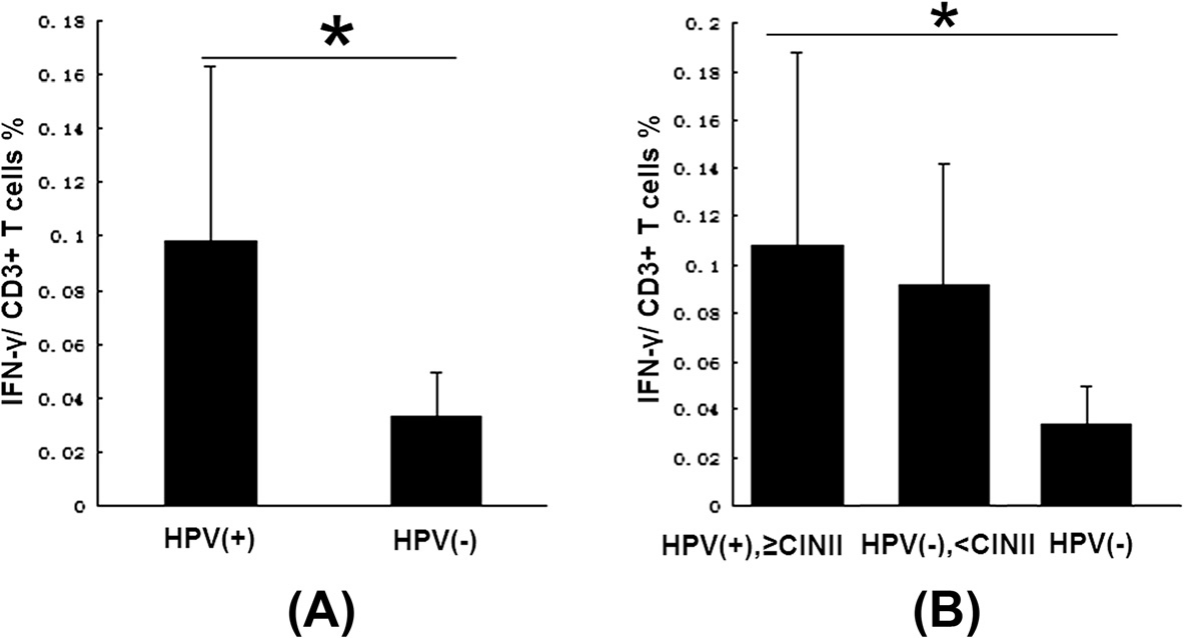
**IFN-**γ **expression in cervical tissues. A**, The bar graph shows RT-PCR results of IFN-γ expression in HPV-positive and HPV-negative cervical tissues. IFN-γ expression is significantly increased in HPV-positive cervical tissues (*p = 0.026). **B**, The bar graph shows IFN-γ expression in ≥ CINII subgroup of HPV-positive cervical tissues, <CINII subgroup of HPV-positive cervical tissues and HPV-negative cervical tissues. IFN-γ expression is significantly increased in the ≥ CINII subgroup of HPV-positive cervical tissues (*p = 0.027), expression in the ≥ CINII subgroup of HPV-positive cervical tissues is similar to that in the < CINII subgroup (*p* = 0.534) and expression in the < CINII subgroup of HPV-positive cervical tissues is comparable to that in the HPV-positive cervical tissues (*p* = 0.078).

INKT cells play a crucial role in the immune response by expressing IFN-γ [19,20]. Whether IFN-γ plays an anti- or a pro-tumor role in human cervical tissues has not been demonstrated. We detected IFN-γ expression to confirm its function and the relationship between iNKT cells and IFN-γ in human cervical tissues. Because the percentages of CD3+ T cells were similar in the HPV-positive and HPV-negative groups, the amount of IFN-γ in cervical samples was measured using the 2^ΔCt^ method, which uses the ratio of the 2^-Ct^ of IFN-γ to the 2^-Ct^ of CD3 in real-time quantitative RT-PCR to prevent interference from other cell types, such as fibroblasts. Increased IFN-γ expression was observed in the HPV-positive group (n = 42) compared to the HPV-negative group (n = 20) (mean, 0.0987 *vs*. 0.0339, *p* = 0.026) (Figure 4A). Furthermore, IFN-γ expression in the high-grade CIN subgroup (n = 15) was increased significantly compared to the HPV-negative group (n = 24) (mean, 0.1081 *vs*. 0.0339, *p* = 0.027) but was similar to expression in the NCT to low-grade CIN subgroup (n = 27) (mean, 0.1081 *vs*. 0.0903, *p* = 0.534). IFN-γ expression in the NCT to low-grade CIN subgroup was similar to expression in the HPV-negative group (mean, 0.0903 *vs*. 0.0339, *p* = 0.078) (Figure 4B).

## Discussion

In this study, we demonstrated that iNKT cells are accumulated in persistently HR-HPV-infected cervical tissues, especially in CINII-III. We further verified that IFN-γ expression was also increased in these HR-HPV-infected cervical tissues, but only in high-grade CIN. There were no significant differences between the HPV-positive and HPV-negative cervical tissues in the distribution of CD3+ T cells. This study is the first to suggest that iNKT cells function to suppress immunity in human persistent HR-HPV-infected high-grade CIN tissues. IFN-γ expression may also be related to HPV infection status.

HPV is the most common sexually transmitted infection worldwide. However, approximately 80% of young women clear HPV infections within 12-18 months [21,22]. Indeed, persistent HR-HPVs, especially HPV types 16/18, are crucial for the progression of high-grade CIN to invasive carcinoma [4,5]. In our study, the total prevalence of HR-HPV infection was 66.7% (134/201), which is consistent with the rate of 75.2% from a recent study of 575 women with abnormal cervical smears at a coloscopy clinic [23]. Moreover, HR-HPV was detected in all 58 women diagnosed as CINII/III. In the HPV-negative group, no CINs were detected, which is consistent with the hypothesis that almost all high-grade lesions are caused by persistent HR-HPV infection [24]. Although HPV infection initiates more than 99% of cervical cancers, only 0.7% of HPV infections will eventually progress to cancer [25]. Because HPV infection alone may not be sufficient for the development of cervical cancer, the majority of the risk of progression to cancer from HR-HPV infection remains unexplained.

The local immune response seems to play an important role in the evolution of CIN. CD3+ T cells were closely correlated with recurrence in patients who underwent conization because of CINIII, which may be due to the failure of the immune response in more severe lesions [26]. In our study, CD3+ T cell numbers were increased in both the epithelium and stromal layers of CINIII cervical tissues, supporting others’ findings that CD3+ T cells may play a causal role in the development of CIN [18]. However, in this study, the distribution and proportion of CD3+ T cells in the HPV-positive group were similar to those in the HPV-negative group, suggesting that HPV infection status was not due to the changes in the infiltration of local CD3+ T cells in cervical tissues. Moreover, in local immunity, it has been reported that the activation of both CD4+ and CD8+ T cells is not altered during the course of HPV infection [14]. These data suggest that although CD3+ T-cell infiltration plays a crucial role in the progression of high-grade CIN, the progression of HPV infection in cervical tissues is not due to changes in CD3+ (including CD4+ and CD8+) T cells.

NKT cells are thought to play a promoting or regulatory role in a wide range of disease conditions [7,8] and are associated with the modulation of NK cells, macrophages and dendritic cells [27,28]. NKT cells also play a role as effector cells, with cytolytic functions against tumor cells [29]. Despite the importance of iNKT cells, little knowledge exists concerning the role of iNKT cells in local immunity. Intratumoral iNKT cell numbers are increased significantly in colorectal carcinomas [30]. In contrast, as in other advanced malignancies [19,20], deficiencies in the number of iNKT cells have been observed in the peripheral blood [31]. It is thought that iNKT cells may migrate from the peripheral blood into tumors. Although we also detected increased numbers of iNKT cells in HPV-positive cervical tissues, another study demonstrated that circulating iNKT cell numbers were not reduced in patients with HPV infections or CINIII patients compared to patients with CINI/II or normal individuals [32]. These data suggest that circulating iNKT cells may be ineffective in the course of HPV infection and the progression of cervical neoplasia, which does not support the anti-tumor hypothesis of migration.

The accumulation of iNKT cells may play a role in suppressing the local immune environment of the cervix. The immunosuppressive role of iNKT cells has been previously reported previously [33,34]. NKT cell-deficient mice with hematopoietic tumors survive longer than wild-type (WT) mice [35]. The same study also showed that donor skin grafts were rejected in iNKT knockout (KO) mice, while the grafts were tolerated in WT mice [12]. During the course from initial HPV infection to the development of histological abnormalities in the cervix, persistent infection is a crucial precondition. In our study, iNKT cells increased in CINII/III lesions, supporting the idea that these high-grade premalignancies have a vital relationship with persistent HR-HPV infection. We hypothesized that the accumulation of iNKT cells induces a local immunosuppressive environment that helps HR-HPV infection to escape from elimination and results in high-grade CIN. Moreover, the number of iNKT cells in the < CINII HPV-positive group was similar to that in the control group, which fits with data demonstrating that low-grade lesions usually clear spontaneously and that HPV infection is usually reversible and may be eliminated by the host immune system without inducing further lesions [36].

Another type of NKT cells, noninvariant NKT cells (type II NKT cells), are considered a suppressive factor in tumor immunosurveillance [37,38]. We did not detect type II NKT cells in our study, but they are not thought to play a role in the local immune response to HPV infection [14].

Furthermore, we sought to investigate how iNKT cells function in cervical tissues. INKT cells produce IFN-γ in several diseases [19,20]. In this study, we found that the amount of IFN-γ increased significantly in HPV-positive cervical tissues, corresponding with iNKT cell accumulation. In general, IFN-γ is a pro-inflammatory cytokine in advanced cancers that promotes anti-tumor immunosurveillance [19,31,39]. In contrast, IFN-γ acts as an anti-inflammatory factor in some autoimmune diseases [40]. A recent murine model indicated that iNKT cells induce a local immune-suppressive environment with IFN-γ production [14], supporting the hypothesis that although low levels of IFN-γ are proinflammatory, once a certain threshold is reached, the anti-inflammatory action of IFN-γ dominates and converts the local epithelial environment into an immune-suppressive setting [41]. Our data indicated that iNKT cells and IFN-γ both exhibit the same increasing trend in HPV-positive cervical tissues. Therefore, we concluded that the accumulation of iNKT cells and the increased production of IFN-γ suppress the persistent HR-HPV-infected cervical environment and induce high-grade CIN.

In addition, IFN-γ expression in < CINII cervical tissues of the HPV-positive group was higher than that in controls but only slightly lower than that in CINII/III cervical tissues, with no statistical significance. We hypothesize that in the early stages of HR-HPV infection, other factors that produce IFN-γ may have little function in CIN progression during persistent HR-HPV infection. However, our study did not confirm a direct relationship between iNKT cell accumulation and IFN-γ expression. To clarify this point, future experiments are needed.

NK cells, as large granular lymphocytes, play an important role in the early stages of immune responses. In addition to cytotoxicity, NK cells function by producing cytokines, especially of IFN-γ [42]. One previous study confirmed that although increased numbers of NK cells were detected in CIN compared to NCT and cervical carcinoma (CxCa), no IFN-γ production by NK cells was detectable in NCT and CIN, except for CxCa [43]. Furthermore, our observations do not exclude the additional involvement of tumor necrosis factor (TNF)-α, IL-17, etc. However, although TNF-α has anti-inflammatory effects, IFN-γ plays a central regulatory role in immune responses [41]; in cutaneous immunity, the secretion of IFN-γ, but not IL-17, played a crucial role [44].

It is possible that different grades of CIN may be present in the different parts of biopsy specimens. In our study, each biopsy sample was divided into two parts for histopathological diagnosis in the pathology department and for laboratory research. As a result, the possibility of grouping bias is inevitable, as the flow cytometry and real-time quantitative RT-PCR destroyed the morphology of the specimens. However, we found that the immunohistochemistry results were consistent with the histopathological grading from the pathology department. Therefore we believe that the grouping bias was at an acceptable level.

In conclusion, the results of this investigation indicate that iNKT cells are capable of suppressing the local immune environment in persistent HR-HPV-infected cervical tissues by producing IFN-γ to induce high-grade CIN. Therefore, blocking iNKT cell accumulation may be useful for either treating persistent HPV infections or preventing the progression from CIN to cervical cancer. Further studies are required to confirm our findings.

## Conclusions

iNKT cell accumulation in cervical tissues during the progression from HPV infection to CIN indicates that iNKT cells may play an important role in suppressing immunity. IFN-γ expression may also be related to the HPV infection status. Preventing the accumulation or functioning of iNKT cells in cervical tissues may be a viable method to prevent the development of CIN.
